# Characterization of the *A1527G* variant of *ABCA7* in an animal model for late‐onset Alzheimer’s disease

**DOI:** 10.1002/alz.091431

**Published:** 2025-01-03

**Authors:** Cristian S Bernabe, Kevin P Kotredes, Ravi S Pandey, Gregory W Carter, Michael Sasner, Adrian L Oblak, Gareth R Howell, Bruce T. Lamb, Paul R Territo, MODEL‐AD consortium

**Affiliations:** ^1^ Indiana University School of Medicine, Indianapolis, IN USA; ^2^ Stark Neurosciences Research Institute, Indianapolis, IN USA; ^3^ The Jackson Laboratory, Bar Harbor, ME USA; ^4^ The Jackson Laboratory for Genomic Medicine, Farmington, CT USA; ^5^ Stark Neurosciences Research Institute, Indiana University School of Medicine, Indianapolis, IN USA

## Abstract

**Background:**

Genome‐wide association studies (GWAS) identified the ATP binding cassette subfamily A member 7 (*ABCA7*) gene as increasing risk for Alzheimer’s disease (AD). ABC proteins transport various molecules across extra and intra‐cellular membranes. *ABCA7* is part of the ABC1 subfamily and is expressed in brain cells including neurons, astrocytes, microglia, endothelial cells and pericytes. However, the mechanisms by which variations in *ABCA7* increase risk for AD are not known.

**Method:**

The IU/JAX/PITT MODEL‐AD Center identified the *A1527G* variant in *ABCA7* (ABCA7*A1527G) as a putative LOAD risk factor. CRISPR/CAS9 was first used to introduce Abca7*A1527G variant to B6.APOE4.Trem2*R47H (LOAD1) mice to assess the transcriptional profiling on brain hemispheres from different ages. The Abca7*A1527G was then incorporated into B6.APOE4.Trem2*R47H.hAb (LOAD2) mice to further evaluate its contribution to LOAD. Female and male LOAD2.Abca7*A1527G and LOAD2 mice were characterized at 4, 12, and 24 months using the following phenotyping pipeline: behavior, PET/CT, multi‐omics, fluid biomarkers, electrophysiology, cognition, and neuropathology.

**Result:**

Brain transcriptional profiling showed that Abca7*A1527G induced changes in gene expression that are similar to some of those observed in human AD (*e.g*., granulocyte/neutrophil migration, and insulin receptor signaling). LOAD2.Abca7*A1527G showed no aging cognitive deficit but did show significant sex‐ and region‐dependent increases in brain glycolysis paralleled by reduced tissue perfusion yielding progressive age‐related uncoupled phenotypes between 4‐12 and 4‐24 months. While multi‐resolution consensus clustering of regional covariance matrices revealed an increase in cluster number and organization in LOAD2.Abca7*A1527G over LOAD2 for both sexes at 4 months, the cluster number and complexity were reduced by 24 months. Importantly, LOAD2.Abca7*A1527G, but not LOAD2, displayed a similar age‐dependent reduction in cluster number for both sexes. Consistent with the uncoupled phenotype, IL6, IL10, and TNFα were elevated in plasma with genotype, but were not age dependent. Conversely, brain levels of IL4, IL12, TNFα, and CXCL1 were decreased, whereas IL2 and IL10 were elevated in LOAD2.Abca7*A1527G relative to LOAD2. Lastly, assessment of plasma levels of Ab40‐Ab42 revealed an age‐dependent increase in both genotypes.

**Conclusion:**

Data collected to date support a model whereby variations in *ABCA7* exert risk for AD through interactions between cerebrovasculature, microglia, and peripheral immune cells.

